# A Molecular Dynamics Simulation Study of In- and Cross-Plane Thermal Conductivity of Bilayer Graphene

**DOI:** 10.3390/ma16206714

**Published:** 2023-10-16

**Authors:** Rafat Mohammadi, Mohammad Reza Ghaderi, Ebrahim Hajian

**Affiliations:** 1 Department of Mechanical Engineering, Faculty of Engineering, Arak University, Arak 38156-88349, Iran; 2 Wood Science and Engineering, Department of Engineering Sciences and Mathematics, Luleå University of Technology, Forskargatan 1, 93187 Skellefteå, Sweden; ebrahim.hajiyan@ltu.se

**Keywords:** bilayer graphene, in-plane thermal conductivity, cross-plane thermal conductivity, non-equilibrium molecular dynamics, anisotropic thermal transport

## Abstract

Efficient thermal management of modern electronics requires the use of thin films with highly anisotropic thermal conductivity. Such films enable the effective dissipation of excess heat along one direction while simultaneously providing thermal insulation along the perpendicular direction. This study employs non-equilibrium molecular dynamics to investigate the thermal conductivity of bilayer graphene (BLG) sheets, examining both in-plane and cross-plane thermal conductivities. The in-plane thermal conductivity of 10 nm × 10 nm BLG with zigzag and armchair edges at room temperature is found to be around 204 W/m·K and 124 W/m·K, respectively. The in-plane thermal conductivity of BLG increases with sheet length. BLG with zigzag edges consistently exhibits 30–40% higher thermal conductivity than BLG with armchair edges. In addition, increasing temperature from 300 K to 600 K decreases the in-plane thermal conductivity of a 10 nm × 10 nm zigzag BLG by about 34%. Similarly, the application of a 12.5% tensile strain induces a 51% reduction in its thermal conductivity compared to the strain-free values. Armchair configurations exhibit similar responses to variations in temperature and strain, but with less sensitivity. Furthermore, the cross-plane thermal conductivity of BLG at 300 K is estimated to be 0.05 W/m·K, significantly lower than the in-plane results. The cross-plane thermal conductance of BLG decreases with increasing temperatures, specifically, at 600 K, its value is almost 16% of that observed at 300 K.

## 1. Introduction

Thermal management at the nanoscale devices is a significant challenge due to the rapid increase in power densities [1,2,3]. For state-of-the-art electronics, having the appropriate thermal conductivity of the material, whether it is high or low, is crucial for thermal management. Development of the next generations of high-power light-emitting diodes (LEDs) and integrated circuits (ICs) demands efficient heat dissipation. This can be achieved using materials with high thermal conductivity to maintain their operational performance and long-term reliability. On the other hand, materials with reduced thermal conductivity are essential for thermoelectric devices, thermal insulators, and even phononic computing devices to ensure a high figure of merit in such applications [4,5].

In recent years, novel materials, especially low-dimensional materials like quasi-one-dimensional nanowires [6,7], nanotubes [8], and two-dimensional (2D) materials [9,10], have attracted significant attention all over the world. Many materials exhibit unique properties in their low-dimensional state that are not observed in their three-dimensional structures. Graphene, the first 2D material discovered via exfoliation in 2004 by Novoselov [11], has exceptional properties in stability, conductivity, and flexibility [12]. Graphene is a single layer of carbon atoms (one atom thick), densely packed in a 2D honeycomb lattice structure which is characterized by sp^2^ hybridization. The strong sp^2^ bonding, combined with the lightweight nature of the carbon atoms in the microscopic structure, imparts to graphene exceptional physiochemical characteristics. These include a high Young’s modulus (~1.0 TPa), a large specific surface area (2630 m^2^/g), and an optical transmittance of 97.7% [13,14,15]. Compared to other mentioned unique features of graphene, its novel thermal properties have gained the most interest. The pioneering experimental measurements, utilizing optothermal Raman method, revealed that single-layer graphene (SLG) could potentially exhibit a thermal conductivity ranging from 2000 to 5000 W/m·K at room temperature [16]. These values surpass the bulk graphite limit of K = 2000 W/m·K for basal planes at room temperature, exceeding even that of a diamond. The remarkable thermal conductivity of graphene arises from the strongest covalent sp^2^ bonds in nature. Experimental research has shown that the thermal transport properties of SLG depend on various factors, such as the geometry size [16,17,18,19], axial strain [20], and functionalization [21].

In addition to SLG, bilayer graphene (BLG) and multilayer graphene sheets also exhibit unique behaviors and possess properties that differ from those of SLG. For example, the room temperature thermal conductivity of few-layer graphene varies from 1300 W/m·K to 2800 W/m·K [22]. Multilayer graphene often exhibits notable thermal conductivity anisotropy, wherein the conductivity perpendicular to the layers (cross-plane) is typically lower than the conductivity along the interfaces (in-plane). Given this anisotropic behavior of graphene, it is necessary to conduct separate investigations into both the in-plane thermal conductivity (k_ip_) and cross-plane thermal conductivity (k_cp_) of BLG and multilayer graphene. While there have been numerous experimental studies on in-plane thermal transport, research on the cross-plane heat transfer of graphene is relatively limited. In a recent study, Sood et al. [23] investigated cross-plane heat transfer in superlattices assembled from single layers of graphene and Mo$S_{2}$, employing a combinatorial experimental approach.

The novel thermal properties of SLG, BLG, and multilayer graphene have been extensively studied by both experimental methods and numerical simulations. While the mentioned experimental research has yielded direct measurements of these properties of graphene, the substantial costs associated with experimentation and the limited ability of nanoscale temperature probing have presented considerable challenges for conducting more extensive investigations. Numerical methods are promising tools to complement experiments and enhance our comprehension of the distinctive thermal properties exhibited by graphene. Molecular dynamic (MD) simulation is one of the main numerical methods for studying the properties of 2D materials, such as graphene. In recent years, there has been a dramatic increase in MD simulations to explore the unique thermal properties of SLG [24,25,26,27,28,29]. References [30,31] provide an extensive review of the latest developments in MD simulation concerning the remarkable thermal properties of graphene.

While there have been numerous studies dedicated to MD simulations of SLG, the attention given to MD simulations of BLG or multilayer graphene is relatively limited. Wei et al. [32] employed the non-equilibrium molecular dynamics method (NEMD) to calculate the k_ip_ of single- and few-layered graphene nanoribbons. Their findings indicated a reduction in the k_ip_ of graphene as the number of layers increased. Cao et al. [33] investigated the k_ip_ of multilayer graphene using NEMD simulations. Their results showed that as the size of multilayer graphene decreased, its k_ip_ also decreased. Guo et al. [34] determined the k_ip_ of interlayer-bonded BLG, tri-layer graphene, and pyrolytic graphite using NEMD. They discovered that interlayer sp^3^ bonding reduces thermal conductivity, with reductions of up to 80%. Additionally, they observed that tensile strain further decreases the thermal conductivity of interlayer-bonded graphene by 50%. Zhan et al. [35] calculated the k_ip_ of a BLG sheet was approximately 64.8 W/m·K, by employing the reverse non-equilibrium molecular dynamics (RNEMD) method. They also observed an 80% reduction in thermal conductivity with a 1.39% increase in bridge defect. Si et al. [36] used NEMD method to study the k_ip_ of both SLG and multilayer graphene sheets, utilizing Tersoff, REBO, and AIREBO potentials. They concluded that for SLG, the optimized Tersoff potential produced the highest thermal conductivity, closely aligning with experimental results. Regarding multilayer graphene, they concluded that the Tersoff potential provides the best simulation of its thermal conductivity. By using the MD method with REBO and Leonard Jones potentials, Li et al. [37] calculated the k_ip_ of misoriented BLG. They explored the effect of interlayer misorientation angle and temperature using NEMD. The study’s conclusion highlighted that thermal conductivities exhibit an approximately linear decrease with the increasing lattice constant of the commensurate misoriented BLG unit cell. Nie et al. [38] examined the k_ip_ and k_cp_ of twisted multilayer graphene sheets utilizing NEMD. The results revealed that the highest thermal conductivity could be achieved when no twist angles were present, for both in-plane and cross-plane conduction. Zhang et al. [39] studied the k_ip_ of BLG by the NEMD, employing different in-layer and interlayer potentials. The Leonard Jones and Kolmogorov–Crespi potentials described the interlayer interactions, while the in-layer interaction potentials, namely Tersoff, REBO, and AIREBO potentials, were compared. Their results showed significant differences in both the magnitude and temperature-related trends of thermal conductivity when different potentials were applied in the simulations.

Reviewing the aforementioned research reveals that, in comparison to the research on in-plane thermal transport, there has been limited focus on investigating the cross-plane conduction at the interface of graphene–graphene. This signifies an existing gap in our understanding that requires resolution. To the best of the authors’ knowledge, a comprehensive investigation into the in-plane and cross-plane conductivity of BLG has been rarely documented in published studies. Therefore, this work aims to explore the thermal conductivity of BLG through NEMD, considering both k_ip_ and k_cp_. Moreover, the effects of several parameters, such as sample dimensions, edge shapes, temperature, and strain, on thermal conductivity are investigated. This study is expected to yield valuable insights for the design and application of BLG in nanoscale devices, particularly where the manipulation of thermal conductivity, whether to suppress it or enhance it, in different directions is essential.

## 2. Simulation Procedures

In this research, we utilized NEMD simulations to calculate the thermal conductivity of BLG. These simulations were executed using the large-scale atomic/molecular massively parallel simulator (LAMMPS) package [40]. Atom visualization was accomplished through the use of the open visualization tool (OVITO) [41]. Moreover, the lattice structure for the input script was provided using visual molecular dynamics (VMD) [42].

**Figure 1:**
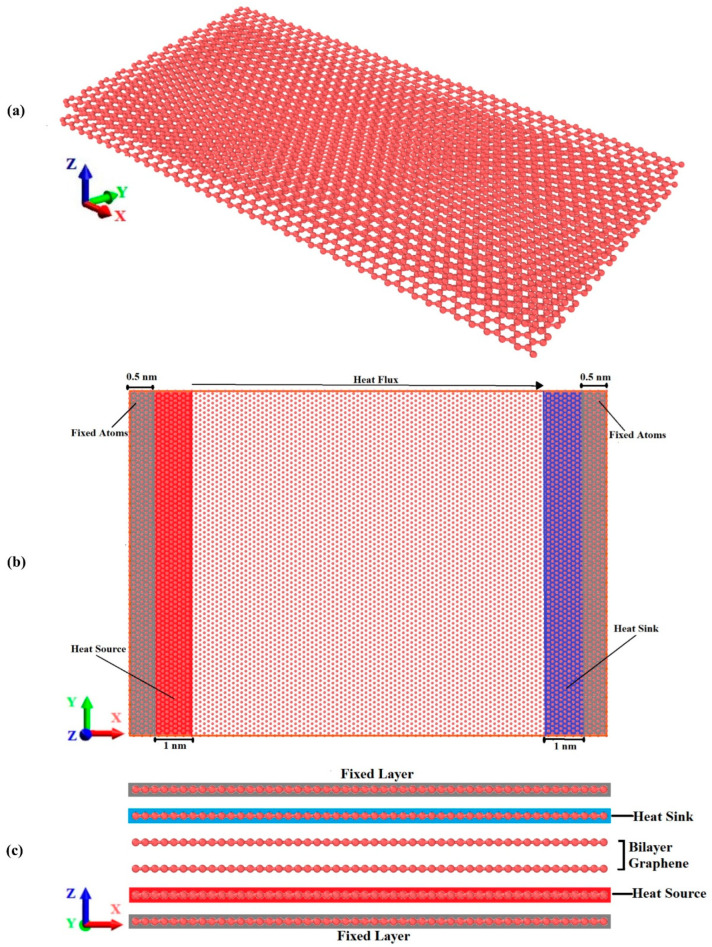
(**a**) Three-dimensional view of BLG sheet. (**b**) Simulation model for the NEMD calculations of the in-plane thermal conductivity. (**c**) Simulation model for the NEMD calculations of the cross-plane thermal conductivity of BLG sheet.

The schematic diagram of BLG is shown in Figure 1a. This figure illustrates the hexagonal structure of zigzag BLG, depicting the relative position of the two layers. Figure 1b illustrates the simulation model for the NEMD simulations of the k_ip_. The simulation domain comprises three regions: the main simulation region, reservoir regions, and fixed walls. Two fixed walls made up of fixed atoms are positioned at the model’s ends in order to prevent atom escape from the system and limit relative sliding motion between graphene layers. Next to these fixed walls, cold and hot reservoirs (heat sink and heat source) are established to create a temperature gradient. Once the simulation structure reaches an equilibrium state, the atom velocities in the reservoir regions are artificially modified to provide a constant heat flux into the main simulation region, situated between the heat source and heat sink. During each time step, the particle velocities within the heat source and heat sink are uniformly scaled, ensuring that a uniform quantity of kinetic energy is consistently added to or deducted from both reservoirs. This process provides a constant heat flux within the simulation domain, establishing a temperature gradient aligned with the direction of heat flow. From the calculated temperature gradient and heat flow, the thermal conductivity of the graphene can be then determined from the Fourier law. To assess the localized temperatures, the model is segmented into 25 equal divisions along the X-axis direction.

To simulate the k_cp_ of the BLG with the NEMD method, the initial configuration of a 6-layer structure needs to be established. The top and bottom layers are set as the fixed layers, and then the heat sink and heat source are positioned as shown in Figure 1c.

The optimized Tersoff potential was employed to describe in-layer interactions [43]. In addition, the van der Waals interaction between distinct layers was modeled using the Lennard–Jones potential, with parameters obtained from Girifalco et al. [44]. The details of the interatomic potentials are provided in Appendix A. These potentials have been proved the most appropriate potentials for modeling the thermal conductivity of multi-layer graphene [38]. Incorporating the Lennard–Jones term into the optimized Tersoff potential to account for interlayer atomic interactions, the optimized Tersoff potential predicts a broader frequency range and a higher value for the high-frequency peak. Furthermore, the graphene’s temperature was determined by calculating the average kinetic energy of every atom within the layer, as indicated in Equation (1):

(1) $$T=\frac{2}{3{\mathrm{Nk}}_{b}}\underset{i}{\sum }\frac{p_{i}^{2}}{2m_{i}}$$

where $k_{b}$ represents the Boltzmann constant, p is the momentum of atoms, m is the mass of atoms, and N represents the number of atoms.

While simulating k_ip_ and k_cp_, the simulation box was first relaxed in an isothermal-isobaric (NPT) ensemble for 100 ps, which proved sufficient to achieve equilibrium. The heat source and heat sink were controlled by Langevin thermostats to maintain constant temperatures. Then, an additional 1 ns was conducted within a microcanonical (NVE) ensemble to ensure the attainment of a desirable linear temperature profile for k_ip_ calculations. For simulating k_cp_, a longer simulation time of 2 ns in the NVE ensemble was necessary to reach a good equilibrium of the temperature profile. This extended duration was required due to the lower thermal conductivity in the cross-plane direction compared to the in-plane direction. Periodic boundary conditions were applied along the X, Y, and Z axis directions.

**Figure 2:**
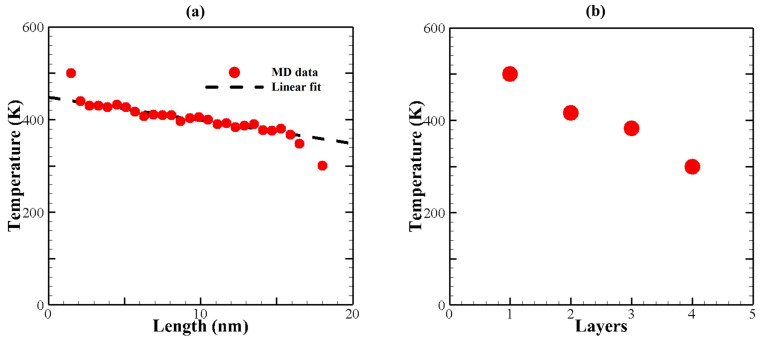
Temperature profiles for NEMD simulation. (**a**) A representative temperature profile for in-plane conduction in the X-direction. (**b**) A representative temperature profile for cross-plane conduction in the Z-direction.

A typical temperature profile of the BLG for the in-plane conduction calculations is depicted in Figure 2a. The temperature gradient was determined using the linear region of this profile. Notably, a temperature jump is observed at the two boundaries. This temperature jump near the heat baths can be attributed to the presence of localized edge modes of phonons [45,46].

The k_ip_ was computed using Fourier law as specified in Equation (2):

(2) $$q=-k_{\mathrm{ip}}A\frac{\partial T}{\partial x}$$

where q is the heat flux obtained from the NEMD simulation, A is the cross-section area, ∂T/∂x is the temperature gradient, and k_ip_ is the in-plane thermal conductivity coefficient.

Figure 2b displays a representative temperature profile for cross-plane calculations in BLG. To determine the cross-plane thermal conductance, Equation (3) was used:

(3) $$G=\frac{q}{A\Delta T}$$

where G represents the cross-plane thermal conductance, which is the inverse of the cross-plane thermal resistance ($R_{\mathrm{cross}})$:

(4) $$R_{\mathrm{cross}}=\frac{1}{G}$$

## 3. Results and Discussion

### 3.1. In-Plane Thermal Conductivity

In order to check the accuracy of the NEMD simulation results for the k_ip_ of BLG, we conducted simulations following the dimensions and operating conditions outlined in the study by Nie et al. [38]. The investigated bilayer zigzag graphene had a size of 22 × 10 nm^2^ with a 0.35 nm interlayer distance. The comparison of results is presented in Table 1 for different temperatures. We performed error analysis using four different heat fluxes across the simulation region. As indicated in Table 1, the k_ip_ calculated in this study exhibits only slight deviations from the findings of Nie et al. [38], with a maximum error of 3.46%.

In addition, comparing the range of k_ip_ obtained for other sizes of BLG, as reported in the upcoming sections, with the values from prior studies, indicates that our NEMD simulation results are consistent with the findings of previous research [36,39].

**Table 1:** Comparisons of the in-plane thermal conductivity of a 22 × 10 nm^2^ BLG sheet between the current study and Nie et al. [38].

Temperature (K)	Nie et al. [38] (W/m·K)	Current study (W/m·K)
200	648 ± 25	653.2 ± 8
300	529 ± 25	520.4 ± 8
400	450 ± 25	465.6 ± 8
500	400 ± 25	406.1 ± 8
600	360 ± 25	368.7 ± 8

In our study, we investigated BLG sheets with dimensions of 10 × 10 nm^2^ and an interlayer distance of 0.335 nm for the calculation of k_ip_. To quantify the impacts of various factors on the k_ip_ of BLG, we varied several key parameters such as length, width, temperature, strain, and edge shapes.

**Figure 3:**
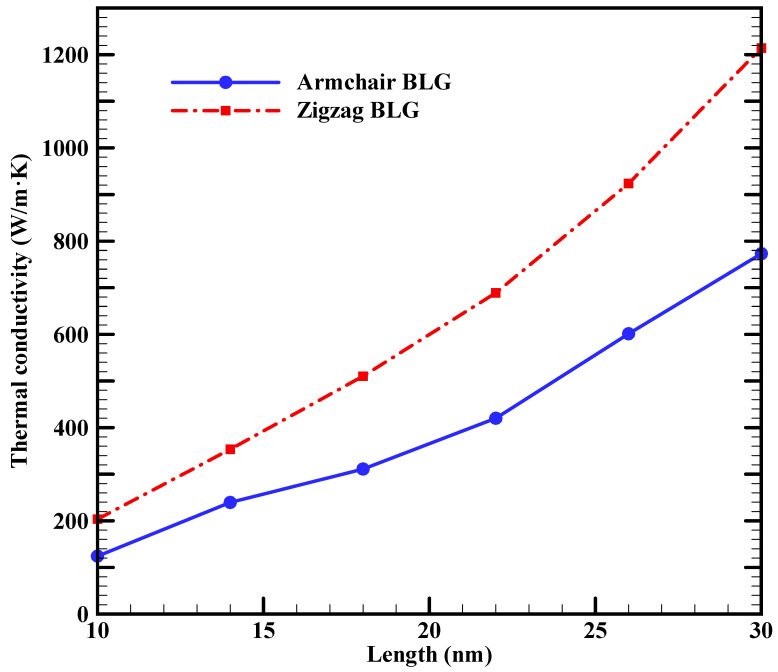
In-plane thermal conductivities of 10 nm wide BLG sheets with armchair and zigzag edges as a function of length at 300 K.

Figure 3 illustrates the influence of sample length on the k_ip_ of BLG with armchair and zigzag edges at 300 K. The thermal conductivities of 10 nm × 10 nm zigzag and armchair BLG are estimated to be 203.6 W/m·K and 124.3 W/m·K, respectively. Contrary to bulk materials, where thermal conductivity is typically size-independent, the results in Figure 3 show that the thermal conductivity of BLG increases with the length of the BLG sheets. The average mean free path of the primary heat carriers, namely acoustic phonons, is in the range of 100–600 nm in graphene [47]. In the present study, the simulation domain is not sufficiently extensive to enable dominant phonons to undergo umklapp scattering. Previous reports indicate that phonons in graphene can travel without scattering, exhibiting ballistic transport [48]. As a result, it is the heat source and the heat sink that ultimately limit the phonon mean free path, leading to the observed increase in thermal conductivity with the sample length. This behavior is in agreement with previously reported experimental findings, where the k_ip_ of SLG was observed to increase with the length of the graphene sheet [19]. The length-dependent thermal conductivity of 2D materials is not limited to cases where the sample size is comparable to the average phonon mean free path; it is observed even when sample lengths significantly exceed the average phonon mean free path [19]. However, for sample lengths exceeding the average phonon mean free path, the phenomenon can be attributed to the 2D nature of phonons in graphene.

Furthermore, Figure 3 shows that the thermal conductivity of the BLG with a zigzag edge is 30–40% higher than that of the armchair edge. Previous studies using various methods, such as MD [24,49,50], Green’s function method [51], or solving the Boltzmann transport equation [52], have shown that the thermal conductivity of graphene with zigzag edges is greater than that of armchair graphene, with reported differences ranging from 15% to 50%. Similar trends have been observed in other 2D nanostructures as well [53,54]. The dependence of thermal conductivity on edge shape in finite-sized graphene sheets can be attributed to several factors, including different phonon scattering rates at the armchair and zigzag edges [49,50,52], edge roughness scattering [52], and phonon localization at edges [24,50]. However, Wang et al. [24] argued that edge roughness scattering should be excluded as the cause for the large thermal conductivity discrepancy in graphene with different edge shapes. They supported their argument with a cross-sectional decomposition of the steady-state heat flux in graphene nanoribbons, revealing a significant suppression of thermal transport at the edges, particularly in armchair configurations. Their phonon spectra analyses and observations of nonuniform heat flux distribution suggested that the strong edge localization of phonons in regions near and at the edges, particularly in armchair edges, is the primary factor responsible for the observed thermal conductivity discrepancy between armchair and zigzag edges [24].

**Figure 4:**
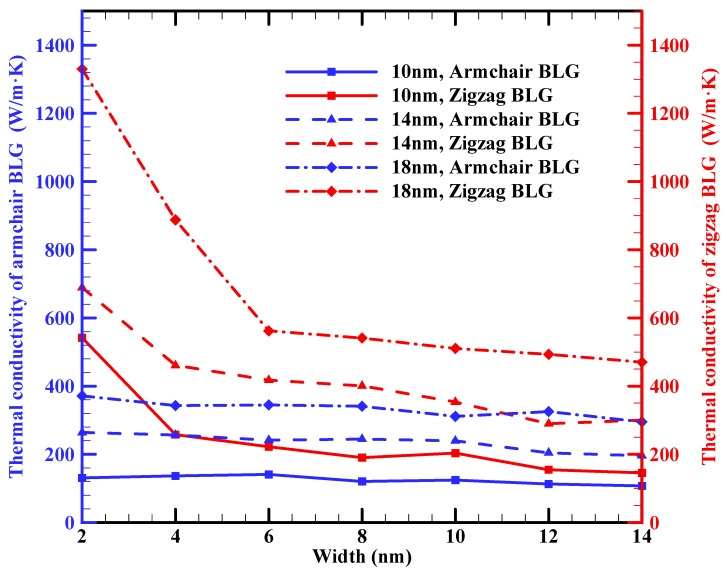
In-plane thermal conductivities of armchair and zigzag BLG sheets with different lengths (10 nm, 14 nm, and 18 nm) as a function of width at 300 K.

Figure 4 illustrates the effect of BLG width on the k_ip_ for three different BLG lengths. It is evident from this figure that the thermal conductivity of BLG sheets with zigzag edges is higher at very small widths, particularly for the smallest BLG lengths (10 nm). This phenomenon could be linked to the limited number of phonons present in the system at these small sizes. This scarcity results in fewer available phonon–phonon combinations that satisfy the momentum and energy conservation rules for scattering, leading to reduced phonon–phonon scattering. The higher thermal conductivity at very small widths aligns with findings from other research [37], where the thermal conductivity of BLG becomes width-independent for widths greater than 6 nm at 300 K.

In addition, Figure 4 reveals that the width of armchair BLG sheets does not exhibit any noticeable effect on their thermal conductivity. These findings are ascribed to the influence of periodic boundary conditions in the simulation, consistent with prior research studies [55].

**Figure 5:**
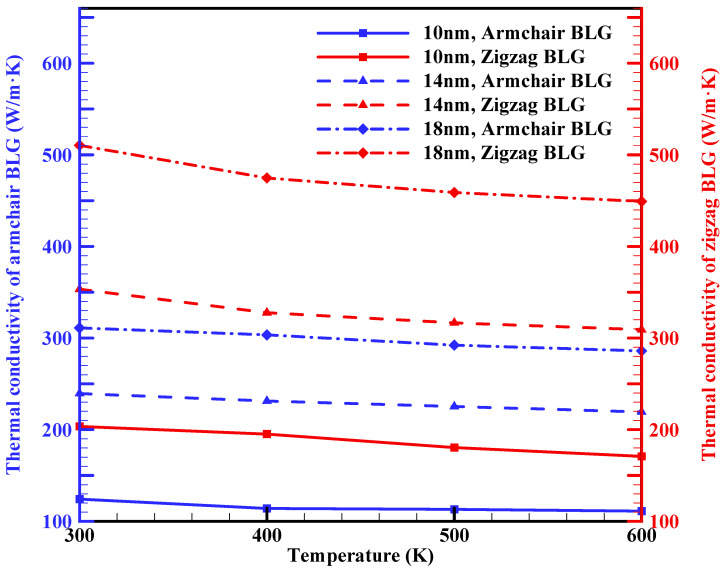
In-plane thermal conductivities of armchair and zigzag BLG sheets with a width of 10 nm and different lengths (10 nm, 14 nm, and 18 nm) as a function of temperature.

Figure 5 presents the effect of temperature variation on the thermal conductivity of zigzag and armchair BLG, covering the temperature range from 300 to 600 K for three different BLG lengths. As it has been proved that MD simulations are not well-suited for accurately predicting the thermal conductivity of graphene with temperature below 300 K [38,56], we focus on temperatures above this threshold. Based on this figure, the thermal conductivity of BLG decreases with increasing temperature, showing a slightly different rate for armchair and zigzag configurations. For a 10 nm× 10 nm sample, the thermal conductivity of the zigzag configuration drops from 203.6 W/m·K to 170.8 W/m·K as the temperature rises from 300 K to 600 K, while for the armchair configuration, it decreases from 124.3 W/m·K to 111.1 W/m·K. The behavior of thermal conductivity for BLG follows a pattern similar to that of bulk dielectric material, decreasing as the temperature rises beyond room temperature. This temperature dependence at high temperatures is attributed to umklapp phonon–phonon scattering. Experimental studies have also addressed the effect of temperature on the thermal conductivity of BLG, with results confirming that BLG’s thermal conductivity decreases as the temperature rises from 300 to 600 K [57,58].

Moreover, as observed in Figure 5, zigzag configurations consistently exhibit higher thermal conductivity than the armchair configurations at all temperatures, as discussed previously.

**Figure 6:**
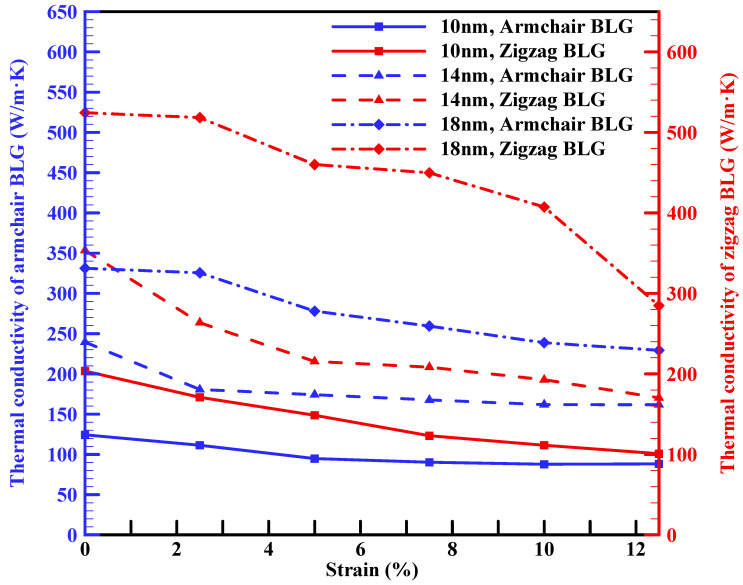
In-plane thermal conductivities of armchair and zigzag BLG sheets with a width of 10 nm and different lengths (10 nm, 14 nm, and 18 nm) as a function of tensile uniaxial strain at 300 K.

Prior research has demonstrated the substantial influence of strain on modulating the thermal conductivity of various 2D materials. The application of strain presents a method to precisely tune the thermal conductivity of materials, as evidenced by these investigations [20,34,59]. Figure 6 illustrates the impact of uniaxial tensile strain on the thermal conductivity of armchair and zigzag BLG sheets. The range of strain considered in this study spans from 0 to 12.5%. This figure demonstrates that increasing strain can result in a decrease in thermal conductivity for both armchair and zigzag configurations. This trend is in good agreement with previous NEMD simulations [59] and experimental studies [20] conducted on SLG confirming the sensitivity of thermal conductivity to tensile strain. The decrease in thermal conductivity with tensile strains can be ascribed to the increase in lattice anharmonicity and the reduction of the stiffness tensor.

Furthermore, it is noteworthy that the thermal conductivity of zigzag BLG demonstrates a higher sensitivity to tensile strain compared to armchair BLG. For a tensile strain of 12.5%, the thermal conductivity reduction is 51% in 10 nm, 52% in 14 nm, and 45% in 18 nm compared to the strain-free values for the zigzag BLG. On the other hand, the thermal conductivity reduction is 29% in 10 nm, 32% in 14 nm, and 30% in 18 nm armchair BLG sheets. This behavior is in agreement with the findings in reference [59], where the thermal conductivity of zigzag graphene nanoribbons exhibited greater sensitivity to the tensile strain compared to armchair graphene nanoribbons. The different sensitivity observed can be attributed to the difference in bond orientation along the direction of loading. Indeed, the effect of tensile strain on the thermal conductivity of graphene with zigzag and armchair edges has been the subject of debate due to the presence of contradictory findings. In another MD simulation, it was observed that the thermal conductivity of armchair graphene nanoribbons exhibited greater sensitivity to tensile strain compared to that of zigzag graphene nanoribbons [60]. This heightened sensitivity was attributed to the greater stress and larger deformation of carbon–carbon bonds along the tensile direction in armchair graphene nanoribbons than zigzag graphene nanoribbons under the same level of strain [60]. Many of these discrepancies can be attributed to computational details, such as the specific interatomic potentials used in simulations and sample size. The true behavior will remain uncertain until relevant experimental results become available.

### 3.2. Cross-Plane Thermal Conductivity

The characteristics of cross-plane thermal conduction differ significantly from those of in-plane conduction in certain materials. For example, in the case of graphite, the k_cp_ has been shown to be two to three orders of magnitude lower than its k_ip_ [61]. The thermal properties of graphene are significantly influenced by those of graphite, given that graphene can retain the anisotropic nature of the graphite crystal. Previous measurements indicate that the cross-plane thermal properties of few-layered graphene are also considerably smaller than its in-plane thermal properties [23,62].

**Figure 7:**
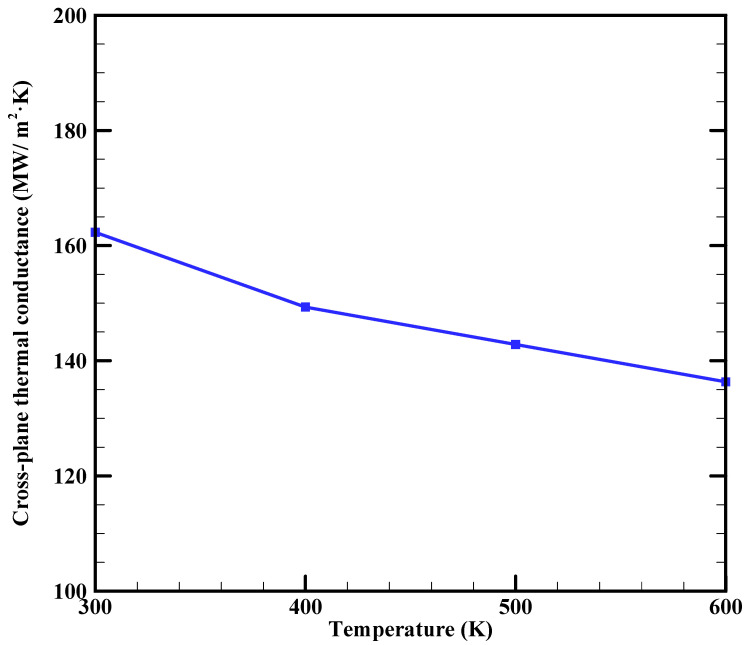
Cross-plane thermal conductance of BLG as a function of temperature.

The effects of temperature on the cross-plane thermal conductance of BLG are illustrated in Figure 7. The graph shows that the cross-plane thermal conductance of BLG at 300 K is 162.3 MW/m^2^·K. To the best of our knowledge, there have been no studies conducted on the thermal conductance of the graphene/graphene interface within BLG. Wei et al. [63] studied the cross-plane thermal resistance in multilayer graphene structures using NEMD simulations, reporting a value of 4.1 × 10^−9^ m^2^·K/W for the cross-plane thermal resistance of 6-layer graphene at 300 K, as shown in Figure 4 of Ref. [63], which corresponds to a cross-plane thermal conductance of 244 MW/m^2^·K. In another research, Ni et al. [64] calculated the cross-plane thermal resistance of 5-layer graphene as 4.83 × 10^−9^ m^2^·K/W using equilibrium molecular dynamics simulations, corresponding to a cross-plane thermal conductance of 207 MW/m^2^·K. In addition, Ding et al. [65] explored interfacial thermal conductance in graphene/MoS_2_ heterostructures using NEMD simulations. For 7-layer graphene, the interfacial thermal conductance at the graphene/graphene interface was calculated as 212.6 MW/m^2^·K, as shown in Table 2 of Ref. [65]. Considering the strong layer number dependence of cross-plane thermal resistance/conductance in multilayer graphene structures and the documented increase in cross-plane thermal conductance with layer number [63], it is reasonable that our obtained cross-plane thermal conductance for BLG is lower than that of 5-, 6-, and 7-layer graphene. It is worth highlighting that when few-layer graphene samples on a substrate are studied, the cross-plane thermal resistance does not significantly change with the number of graphene layers [23,62], indicating that the thermal resistance between graphene and its substrate has a more notable effect than the resistance between individual graphene sheets.

Our simulation results reveal that the corresponding k_cp_ of BLG at 300 K is 0.054 W/m·K, which closely aligns with both the reported values of 0.05 W/m·K for BLG (Figure 5 of Ref. [66]) and 0.07 W/m·K for 6-layer graphene [63]. These findings highlight that the k_cp_ is significantly lower than the in-plane results by approximately four orders of magnitude. This anisotropic behavior arises from the high k_ip_ of graphene, which is ascribed to the strong covalent sp^2^ bonding between adjacent carbon atoms. This type of bonding is known to be one of the strongest in nature, marginally stronger than the sp^3^ bonds in diamond. In contrast, the adjacent graphene layers in multilayer graphene are linked by weak van der Waals interactions.

Furthermore, Figure 7 demonstrates that the cross-plane thermal conductance of BLG decreases with rising temperatures in the range of 300 to 600 K. This trend suggests that temperature impacts the cross-plane thermal conductance similarly to the k_ip_, which monotonically decreases with increasing temperature. This observation aligns qualitatively with prior research on the temperature effect on cross-plane heat transfer in few-layered graphene [63,66,67]. The underlying physics behind this phenomenon lies in the dominance of phonon scattering in graphene heat transfer. Consequently, heat transfer should decrease as the temperature rises, independent of the direction of heat transport. In addition, it is evident from Figure 7 that the temperature-induced reduction is not particularly pronounced: the thermal conductance at 600 K is approximately 16% of that at 300 K, consistent with the behavior reported for 5-layer graphene, where cross-plane resistance was found to be almost unaffected by temperature [64].

## 4. Conclusions

This study investigated the in-plane and cross-plane thermal conductivity of BLG sheets using NEMD. We employed the optimized Tersoff potential to describe in-layer interactions, while the van der Waals interaction between different layers was modeled using the Lennard–Jones potential. The obtained results for both k_ip_ and k_cp_ of BLG sheets exhibited strong agreement with published data. Furthermore, we conducted a parametric study and thoroughly analyzed the resulting outcomes.

Our results showed that the k_ip_ of 10 nm × 10 nm zigzag and armchair BLG sheets at room temperature are around 203.6 W/m·K and 124.3 W/m·K, respectively. The k_ip_ of BLG increased with the length of the sheets: for 10 nm × 30 nm sheets, the values for the zigzag and armchair configurations increased to 1214.15 W/m·K and 773.15 W/m·K, respectively. The influence of sheet width, due to the applied periodic boundary conditions, was less evident on BLG’s thermal conductivity. In addition, the k_ip_ of BLG decreased with increasing temperature, with a slightly divergent rate observed between armchair and zigzag configurations. A 10 nm × 10 nm zigzag BLG sample, for instance, saw a reduction from 203.6 W/m·K to 170.8 W/m·K as the temperature increased from 300 K to 600 K. Conversely, the armchair configuration’s thermal conductivity decreased from 124.3 W/m·K to 111.1 W/m·K over the same temperature range. Furthermore, applying uniaxial tensile strain on the BLG sheet led to a reduction in thermal conductivity for both armchair and zigzag configurations, with zigzag BLG displaying a higher sensitivity compared to armchair BLG. A tensile strain of 12.5% on a 10 nm × 10 nm sample resulted in a thermal conductivity reduction of 51% for zigzag BLG and 29% for armchair BLG when compared to strain-free values.

Our findings also demonstrated that the cross-plane thermal conductance of BLG at 300 K is 162.3 MW/m^2^·K with the corresponding k_cp_ of 0.054 W/m·K. These findings highlighted that the k_cp_ is significantly lower than the in-plane results by about four orders of magnitude. As temperatures increased in the range of 300 to 600 K, the cross-plane thermal conductance exhibited a reduction. However, the temperature-induced reduction was not highly pronounced and the thermal conductance at 600 K was approximately 16% of that at 300 K.

The high anisotropic heat conduction properties observed in BLG sheets hold promise for applications in the thermal management of nanoscale devices that require controlled regulation of thermal conductivity in different directions.

## Data Availability

All data are included in this paper.

## Appendix A. Interatomic Potentials

According to the Tersoff equation, the pair potential (U_ij_), comprises contributions from both attractive ($f_{\mathrm{ij}}^{A})$ and repulsive ($f_{\mathrm{ij}}^{R}$) interactions between each unique atomic pair i-j, at a distance r_ij_:

(A1) $$U_{\mathrm{ij}}=f_{\mathrm{ij}}^{c}\left(a_{\mathrm{ij}}f_{\mathrm{ij}}^{R}-b_{\mathrm{ij}}f_{\mathrm{ij}}^{A}\right)$$

The function $f_{\mathrm{ij}}^{c}$ is the cut-off term that limits the interaction range to the nearest neighbors. The repulsive and attractive contributions to the bond energy of i-j pair decrease exponentially with the separation distance r_ij_. These contributions are expressed as:

(A2) $$f_{\mathrm{ij}}^{R}=A_{T}e^{-\lambda_{1}r_{\mathrm{ij}}}$$

(A3) $$f_{\mathrm{ij}}^{A}=B_{T}e^{-\lambda_{2}r_{\mathrm{ij}}}$$

The term a_ij_ in Equation (A1) acts as a range-limiting term for the repulsive potential and is commonly set to a value of 1. On the other hand, the bond angle term (b_ij_) is influenced by the local coordination of atoms surrounding atom i, and the angle between atoms i, j, and k ($\theta_{\mathrm{ijk}}$):

(A4) $$b_{\mathrm{ij}}={\left(1+\beta^{n}\zeta_{\mathrm{ij}}^{n}\right)}^{\frac{-1}{2n}}$$

(A5) $$\zeta_{\mathrm{ij}}^{n}=\underset{k\neq i.j}{\sum }f_{\mathrm{ik}}^{C}{\;g}_{\mathrm{ijk}}e^{\left[\lambda_{3}^{3}\;{\left(r_{\mathrm{ij}}-r_{\mathrm{ik}}\right)}^{3}\right]}$$

(A6) $$g_{\mathrm{ijk}}=1+\frac{c^{2}}{d^{2}}-\frac{c^{2}}{d^{2}+\left(\cos(\theta_{\mathrm{ijk}}\right)-h{)}^{2}}$$

The parameters for the optimized Tersoff potential are obtained from Ref. [43] and are presented in Table A1.

**Table A1:** Parameters of the optimized Tersoff potential for carbon-based systems [43].

Parameter	Value	Parameter	Value
$A_{T}$ (eV)	1393.6	n	0.72751
$B_{T}$ (eV)	430.0	R (Å)	1.95
c	38,049.0	β	1.5724 × 10^−7^
d	4.3484	$\lambda_{1}$ (1/Å)	3.4879
D (Å)	0.15	$\lambda_{2}$ (1/Å)	2.2119
h	−0.930	$\lambda_{3}$ (1/Å)	0.0000

The van der Waals interaction between layers was modeled using the Lennard-Jones potential, as represented in Equation (A7):

(A7) $$U=4\varepsilon \left[-{\left(\frac{\sigma }{x}\right)}^{6}+{\left(\frac{\sigma }{x}\right)}^{12}\right]$$

Here, ε and σ correspond to the Lennard-Jones parameters, while x is the interatomic distance. The ε parameter characterizes the well depth, which determines the strength of interaction between two particles, whereas σ represents the distance at which the potential energy becomes zero between the two particles. The equilibrium distance (x_0_) is determined by:

(A8) $$x_{0}=2^{\frac{1}{6}}\;\sigma ={\left(\frac{2B_{\mathrm{LJ}}}{A_{\mathrm{LJ}}}\right)}^{\frac{1}{6}}$$

and the well depth is given by:

(A9) $$\varepsilon =\frac{A_{\mathrm{LJ}}{}^{2}}{4B_{\mathrm{LJ}}}$$

where $A_{\mathrm{LJ}}$ and $B_{\mathrm{LJ}}$ are the attractive and repulsive constants in the Lennard-Jones potential. These parameters are derived from the work of Girifalco et al. [44] and are presented in Table A2.

**Table A2:** Parameters of the Lennard-Jones potential for graphene-graphene interactions [44].

Parameter	$A_{\mathrm{LJ}}$ $\;(\mathrm{eV}\times {Å}^{6})$	$B_{\mathrm{LJ}}$ $\;(\mathrm{eV}\times {Å}^{12})$	ε (eV)	σ (Å)
Value	15.12	24.1 × 10^3^	2.39 × 10^−3^	3.41
